# Air traffic control forgetting prediction based on eye movement information and hybrid neural network

**DOI:** 10.1038/s41598-023-40406-z

**Published:** 2023-08-11

**Authors:** Huibin Jin, Weipeng Gao, Kun Li, Mingjian Chu

**Affiliations:** 1 College of Transportation Science and Engineering, Civil Aviation University of China, Tianjin, 300300 China; 2 College of Safety Science and Engineering, Civil Aviation University of China, Tianjin, 300300 China; 3 School of Civil and Transportation Engineering, Hebei University of Technology, Tianjin, 300401 China; 4 COMAC Shanghai Aircraft Design and Research Institute, Shanghai, 201210 China

**Keywords:** Human behaviour, Engineering, Mathematics and computing

## Abstract

Control forgetting accounts for most of the current unsafe incidents. In the research field of radar surveillance control, how to avoid control forgetting to ensure the safety of flights is becoming a hot issue which attracts more and more attention. Meanwhile, aviation safety is substantially influenced by the way of eye movement. The exact relation of control forgetting with eye movement, however, still remains puzzling. Motivated by this, a control forgetting prediction method is proposed based on the combination of Convolutional Neural Networks and Long-Short Term Memory (CNN-LSTM). In this model, the eye movement characteristics are classified in terms of whether they are time-related, and then regulatory forgetting can be predicted by virtue of CNN-LSTM. The effectiveness of the method is verified by carrying out simulation experiments of eye movement during flight control. Results show that the prediction accuracy of this method is up to 79.2%, which is substantially higher than that of Binary Logistic Regression, CNN and LSTM (71.3%, 74.6%, and 75.1% respectively). This work tries to explore an innovative way to associate control forgetting with eye movement, so as to guarantee the safety of civil aviation.

## Introduction

In recent years, the number of civil aviation control flights has increased rapidly^1^. The air traffic controller (hereinafter referred to as the controller), as the regulator of air traffic order, should undoubtedly endure the huge pressure from increasing flight tasks^2^. Thus, the controllers are more likely to make human errors threatening aviation safety in the process of control work^3,4^. Studies have shown that control forgetting is the most adverse factor, which is commonly observed all over the world.

Vision is the main way to monitor the status of aircrafts. Appropriate selection and processing of visual information is an indispensable cognitive function for the controller^5^. A large number of qualitative and quantitative studies on eye movement behavior data in ergonomics research have shown that operators’ focus can be assessed by eye movement characteristics such as the form of eye movement, blink frequency, saccade speed, and pupil diameter^6–8^. Moreover, working status such as degree, fatigue, and cognitive load is important and effective to control radar surveillance, which is essentially a complicated human–computer interaction process^7^. Therefore, it is naturally to explore an innovative way to predict control forgetting behavior by analyzing the eye movement characteristics of the controller.

In recent years, many researchers have carried out a lot of research on eye movement characteristics of controllers. It is well known that eye movement indicators can be used to characterize the fatigue state of workers. Based on the advantages of non-invasiveness of eye movement, Jin et al. conducted an experiment to detect the fatigue state of controllers in real time^9,10^, and found that the controllers’ perclos, fixation point, saccade speed, pupil diameter and etc. can effectively characterize the fatigue state of the controller. Moreover, Palma Fraga et al.^11^ explored the visual search and conflict mitigation strategies used by expert air traffic controllers through eye movement features. Lanini-Maggi et al.^12^ used the controllers’ eye movement indicators to evaluate how visual search entropy and participation can predict the performance of multi-target tracking air traffic control tasks. It can be seen that the research of eye movement characteristics in the working status of controllers is not only limited to exploring the relationship between eye movement data and controllers under different scenarios, but also focused on the usage of eye movement characteristics to judge the status of the controller, to predict the behavior of the controller, and to ensure aviation safety. However, for more complicated scenarios and more comprehensive analysis, it is necessary and important to use multiple eye movement indicators^13^ and to mine deep characteristic rule of the eye movement data^14^.

With the development of deep learning and machine learning, some innovative methods such as convolutional neural network (CNN) and recurrent neural network (RNN) are widely used to identify the working status of workers^15–18^. Yet, the application of neural networks and deep learning methods to process and recognize the eye movement signals of controllers is still an open issue. Better recognition performance can facilitate control efficiency. In addition, in the existing research, the timing characteristics of the controller's eye movement signals have not been fully explored.

Taken overall, understanding the eye movement features of the controller more deeply can improve the accuracy of identifying the status of the controller through the eye movement feature. Motivated by this, based on an eye movement experiment, we propose an innovative method using CNN-LSTM to predict aviation control forgetting. By virtue of the new recognition method, the accuracy of the recognition of the controller's eye movement characteristics and status is effectively improved, indicating that this method is conducive to better prediction of possible control forgetting events, thereby ensuring the safety of civil aviation.

## Experiment method and data processing

### Ethics approval and consent to participate

All experimental protocols has been reviewed and approved by the Academic and Ethics Committee at General Aviation College of CAUC (Civil Aviation University of China). It will not involve trade secrets, conforms to ethical principles and relevant national regulations. All experimental methods were carried out in accordance with relevant guidelines and regulations, and all research has been performed in accordance with the Declaration of Helsinki. All subjects have provided the written informed consent.

### Participants

In order to collect the corresponding eye movement data, 22 junior students from the Civil Aviation University of China, majoring in control, were recruited for this experiment. All of these subjects have a certain basic knowledge of control. Among the 22 subjects, there were 12 males and 10 females, ranging in age from 19 to 22, with an average value of 20.3 ± 2.4. All subjects voluntarily participate in this regulatory simulation experiment and will receive corresponding rewards.

### Experimental scenario design

The radar interface in the radar control simulation experiment consists of two parts, namely the sector interface and operation interface. The sector interface is mainly composed of four exits and two airports. When the experiment starts, a certain number of aircrafts with a specific speed and altitude will appear in the sector, and each tries to fly to the corresponding destination. The operation interface is mainly composed of a direction control area, a speed control area and an altitude control area, which respectively control the direction, speed and altitude of the aircrafts respectively.

When the simulation control experiment starts, several aircrafts will be randomly generated from the four exits of the sector interface. These aircraft will maintain a fixed forward direction, flight speed and operating altitude without being controlled. The operator clicks on the corresponding aircraft to select it with the mouse, and then makes appropriate adjustments to its forward direction, flight speed, and operating altitude on the operation interface to ensure that the aircraft reaches the corresponding destination smoothly. In this process, they should try their best to avoid aircraft collisions, wall collisions, collision fields and other control errors.

**Figure 1:**
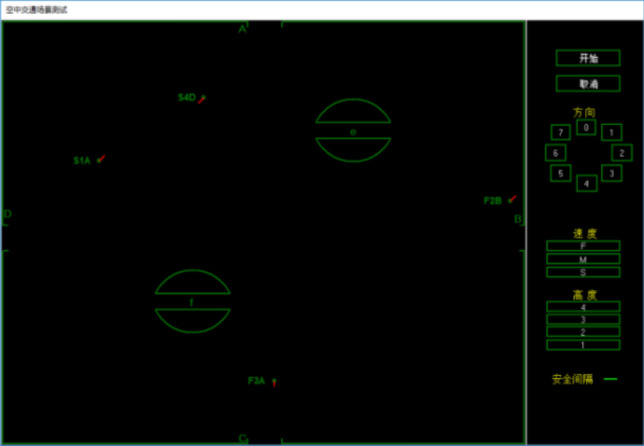
Simulated experiment interface.

The interface of this experiment is similar to the real radar interface. The aircraft targets in the sector are displayed in the main interface with a refresh frequency of 1 s, instead of moving smoothly and continuously in the interface. The experiment interface is shown in Fig. 1.

### Experimental process and data collection

This simulation control experiment uses the German SMI head-mounted eye tracker to collect the eye movement data of the subjects, and the sampling frequency is 100 Hz. The simulation control experiment process is as follows:

1. Before the experiment starts, the participants are informed of the purpose, content and operation requirements of the experiment. Then, the participants do some exercises to familiarize themselves with the operation process. When the operation accuracy rate of the participants reaches more than 90%, they are considered to be able to participate formally experiment.
2. Participants sit in front of the experiment screen, adjust the height of the seat to ensure a comfortable sitting posture, and appropriately adjust the height of the experiment screen so that they can look up at the screen.
3. After the participants put on the eye tracker, a calibration test is required to ensure the accuracy of the collected data.
4. The duration of each experiment is 90 min.

### Data processing

The original eye movement data collected by the eye tracker includes the subject’s pupil diameter, timestamp, confidence, and horizontal and vertical coordinates of the line of sight.

**Figure 2:**
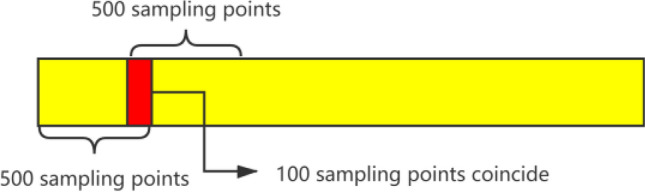
Truncated sliding window.

Before processing the eye movement features, the original eye movement data needs to be preprocessed. Here, we use linear interpolation to fill in the pupil diameter data lost due to blinking, and use the average value around the missing data to fill in the empty data caused by improper collection. After processing the original eye movement data, it is truncated by a sliding window. The window length is 500 sampling points (5 s), and adjacent windows overlap 100 sampling points, as shown in Fig. 2. In fact, the sliding window with 500 sampling points is found most suited for the control simulation experiment used through multiple experiments, since the participants were reminded and reacted to the control forgetting, and the conflict could be resolved in about 5 s. In this sense, the eye movement characteristics and control behavior of the subject can be regarded as an event which lasts 5 s.

By further analyzing the original eye movement data, in the light of Ref.^19^, a total of 44 eye movement features, including total number of fixation points, blink rate, total length of saccades, and so on, are obtained. According to whether they are related to the time series^19^, the 44 eye movement features are divided into two types: time-related eye movement features (23 eye-movement metrics, see Table 1) and time-independent eye movement features (21 eye-movement metrics, see Table 2). For further analysis the 44-dimensional data is denoted by $X_{{{\text{base}}}} = [X_{{{\text{tc}}}} ,X_{{{\text{tic}}}} ]$, where $X_{{{\text{tc}}}}$ refers to time-related eye movement characteristic data, and $X_{{{\text{tic}}}}$ represents time-independent eye movement characteristics data.

**Table 1:** Time-related eye movement characteristics.

Eye-movement metrics	Category
1. Total number of gaze points	Fixation category
2. Number of gaze points per second	Fixation category
3. Average gaze duration	Fixation category
4. Gaze width	Fixation category
5. Nearest neighbor distance	Fixation category
6. Total gaze time	Fixation category
7. Maximum gaze time	Fixation category
8. Gaze density	Fixation category
9. Number of blinks	Blink category
10. Blink rate	Blink category
11. Longest blink depth	Blink category
12. Current blink depth	Blink category
13. Closed eye time	Blink category
14. Total number of saccades	Saccades category
15. Total length of saccades	Saccades category
16. Maximum saccade distance	Saccades category
17. Average saccade length	Saccades category
18. Total saccade time	Saccades category
19. Average saccade time	Saccades category
20. Maximum saccade time	Saccades category
21. Average saccade speed	Saccades category
22. Overall saccade speed	Saccades category
23. Pupil diameter	Other

**Table 2:** Time-independent eye movement characteristics.

Eye-movement metrics	Category
1.Median fixation duration	Fixation category
2. Fixation time ratio	Fixation category
3. Fixation point distribution standard deviation	Fixation category
4. Horizontal range	Fixation category
5. Fixation time standard deviation	Fixation category
6. Fixation time skewness	Fixation category
7. Fixation time kurtosis	Fixation category
8. Location of first fixation	Fixation category
9. Standard deviation of the number of blinks	Blink category
10. Noting	Blink category
11. Returning	Blink category
12. Arithmetic mean center	Saccades category
13. Weighted average center	Saccades category
14. Vertical range	Saccades category
15. Median saccade distance	Saccades category
16. Standard deviation of saccade distances	Saccades category
17. Skewness of saccade distance	Saccades category
18. Kurtosis of saccade distance	Saccades category
19. Scan-path length	Saccades category
20. Global/local ratio	Saccades category
21. Dispersion of saccades	Saccades category

Considering that the numerical value ranges of different eye movement features are quite different, if these data are directly input into the model for training, it is likely that the numerical span is too large and will adversely affect the convergence of the model. Therefore, this article uses the Min–Max standardized method to process the data. For a certain feature $X$, each data $x \in X$ in it is mapped by the following formula:

1 $$ x^{\prime} = (x - \min (X))/(\max (X) - \min (X)) $$

## Methodology

**Figure 3:**
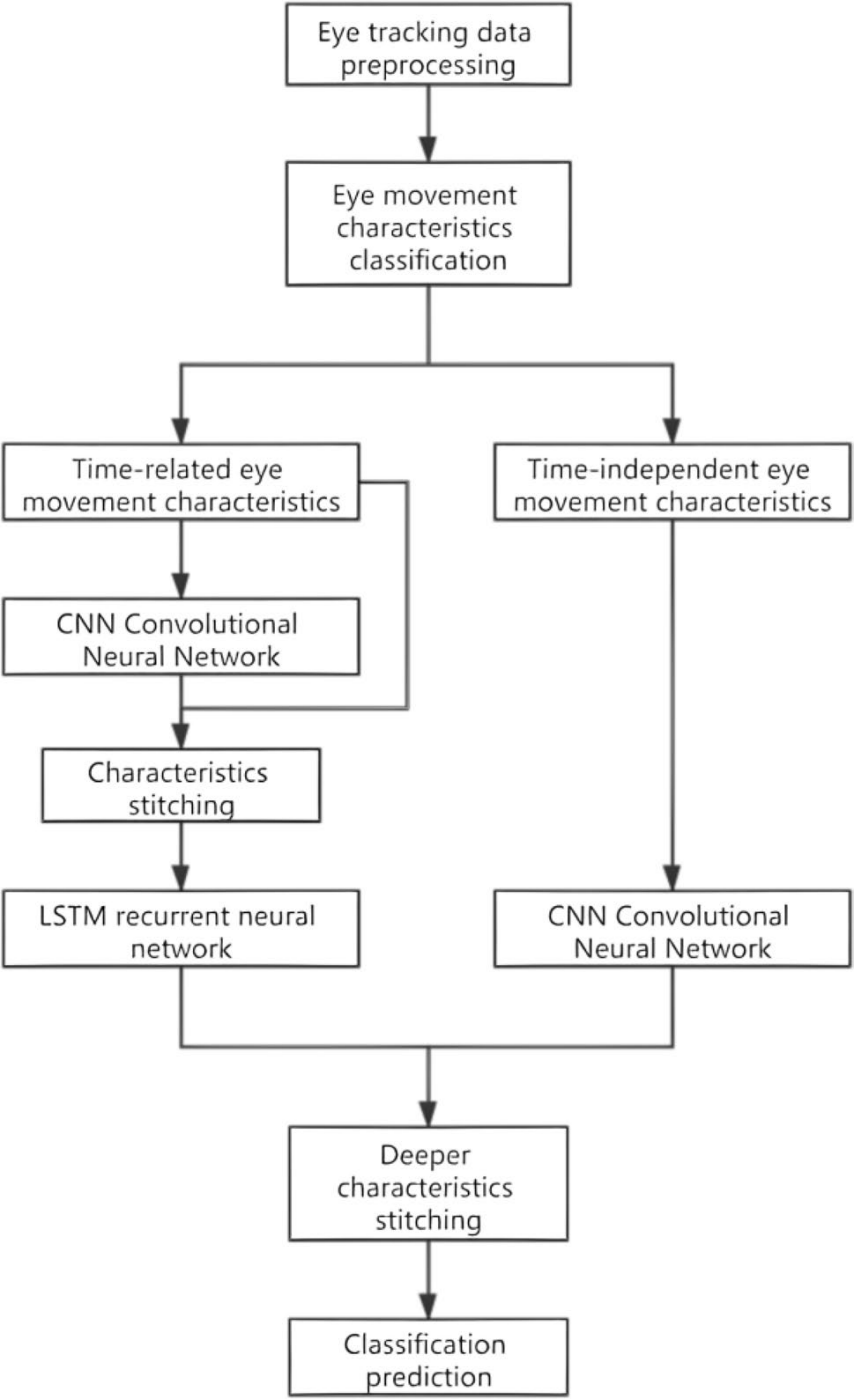
The basic framework of the methodology.

This method is mainly based on whether the controller’s eye movement features are time-related or not. Experimental data is first divided into time-related eye movement features and time-independent eye movement features, and then LSTM neural network and CNN neural network are used to process the two features in order to obtain two types of feature representation. Subsequently, timing-related data from CNN-LSTM module and timing-independent data from CNN module is spliced for final classification. The basic framework of the methodology is shown in Fig. 3.

### Related parameter setting and input data classification

After normalizing all eye movement feature sequences, iterative training is performed according to 64 event sequences as a batch. As a hyperparameter, the size of the batch is positively related to the size of the required memory space. At the same time, the larger the batch value also means the faster the training speed and the more comprehensive features extracted^19^. According to many experimental tests carried out before, this article sets the value of the training batch to 64 to achieve the best results^19^.

In terms of sequence length, if the sequence is too short, the model cannot extract enough time sequence information due to insufficient time span; if the sequence is too long, it is easy to lose the before and after correlations^20^. Here, through the traversal test method, we derive the optimal sequence length value 6, that is, every 6 events are extracted as an event sequence through a sliding window.

**Figure 4:**
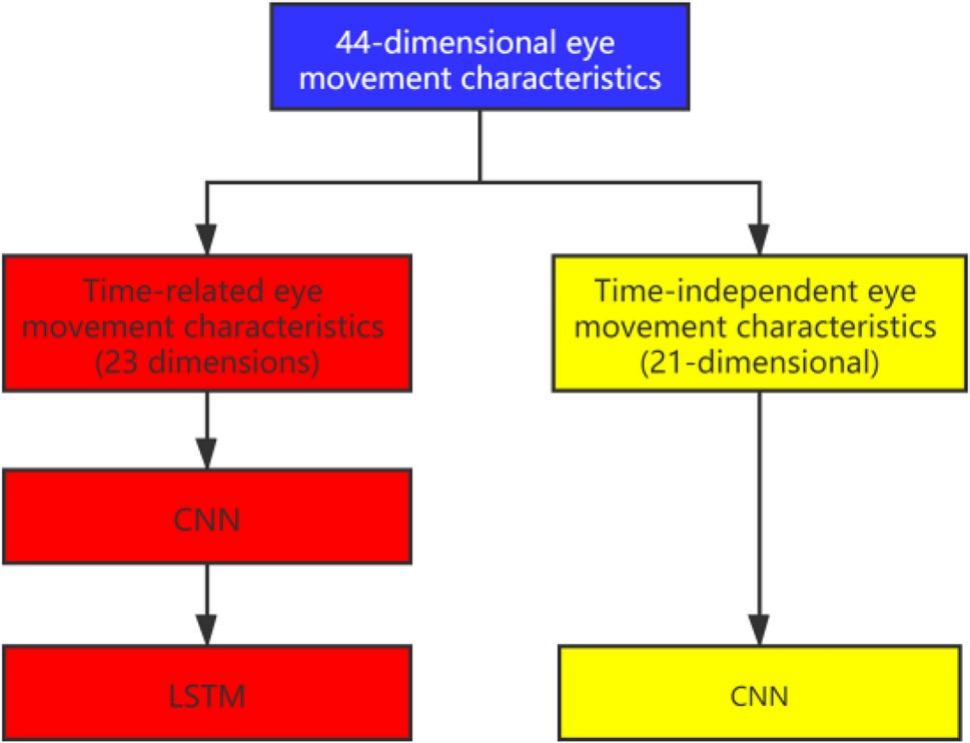
Different eye movement feature processing methods.

As shown in Fig. 4 the input data of this model is divided into two parts, namely time-related eye movement features and time-independent eye movement features. For timing-related eye movement features, CNN is first used to extract more advanced and abstract features, and then these features outputted by CNN are handled by LSTM, which is particularly suitable for time series; while for timing-independent features, CNN is used to process them directly.

### Time series related eye movement data processing method

#### CNN module

**Figure 5:**
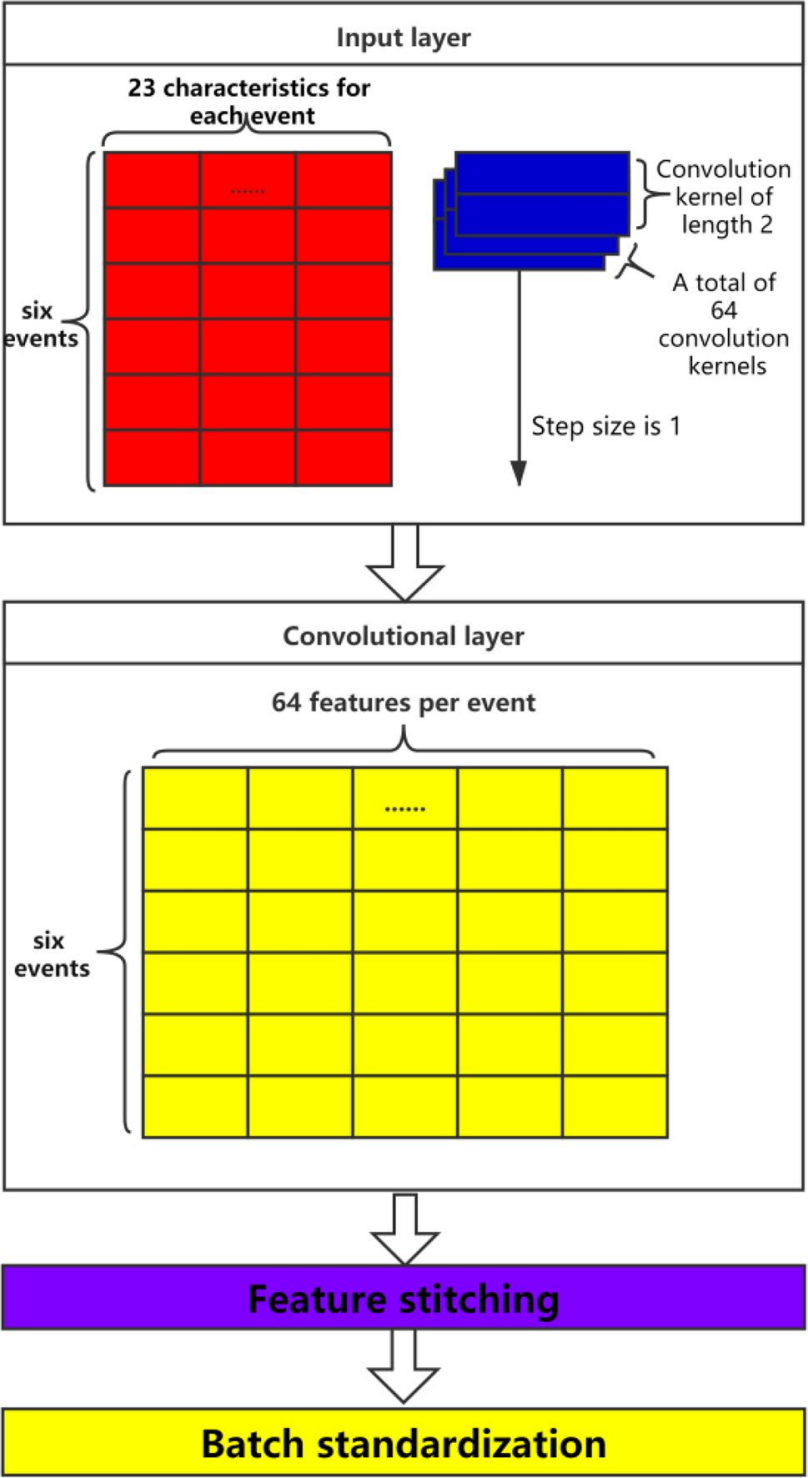
CNN module.

When using the CNN-LSTM combined model to process timing-related eye movement features, $X_{{{\text{tc}}}}$ in $X_{{{\text{base}}}}$ is used as the bottom input of the network, and CNN is used to extract more advanced and abstract features. As described in Section “Data processing”, the eye movement characteristics and control behavior of the subjects can be regarded as an event. Here, as shown in Fig. 5, there are 3 convolutional layers in CNN. Length 2 represents a matrix with 2*2 convolution kernel, and each event has 23 features.

Data imported into CNN is not single or independent, but batch import. Here 6 rows (6 events) and 23 temporal correlation features are used to form a 6 × 23 event sequence matrix. During CNN process, 64 groups of 6 × 23 matrices are imported simultaneously to form a dimension matrix of 64 × 6 × 23. It is worth noting that although the convolution kernel of CNN here is two-dimensional, its horizontal dimension is actually fixed. We only need to specify the vertical length of the convolution kernel, so the convolution kernel can be equivalent to a one-dimensional volume. Convolution kernel and convolution only need to move along the longitudinal dimension.

In this article, 64 convolution kernels are defined, so 64 different features can be extracted in the first layer of the network. Therefore, the output feature size of CNN is 64 × 6 × 64. These three indexes represent the training batch size, event sequence length, and 64 high-level features, respectively. The feature data obtained through CNN neural network is denoted by $X_{deeper}$. After the convolution operation, Re LU is used as the activation function^21^.

Taking $x_{tc}^{i}$ as the *i*-th sample of the input, the process can be expressed by the following formula:

2 $$ C(x_{tc}^{i} ) = x_{depper}^{i} $$

3 $$ C( \cdot ) = {\text{Re}} LU(BN(Conv(x_{tc}^{i} ,W))), $$

where $C( \cdot )$ is a function for extracting high-level features, $Conv( \cdot )$ means convolution operation, *W* refers to the parameters in convolution calculation, and $x_{depper}^{i}$ is the deep feature extracted from the *i*-th sample.

**Figure 6:**
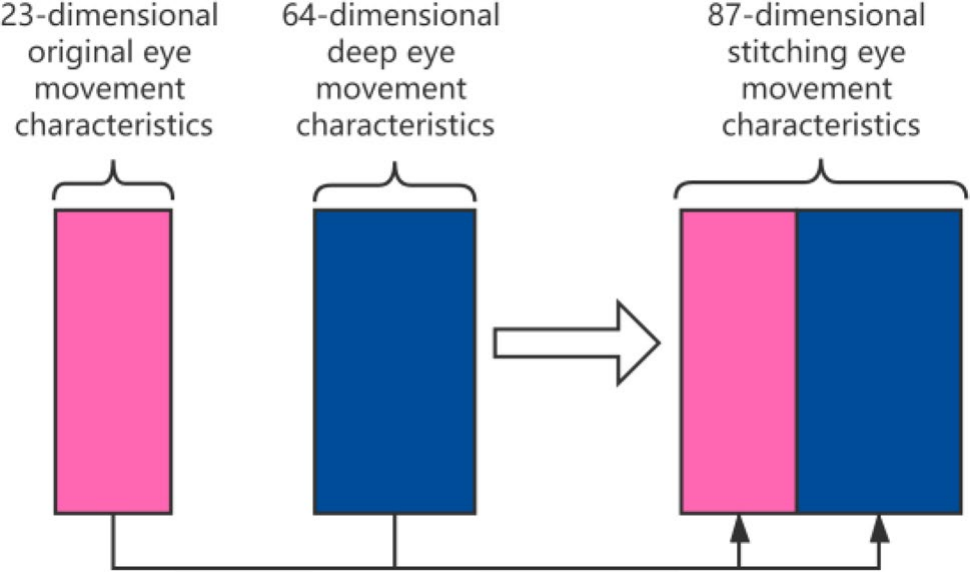
Eye movement feature stitching.

In order to retain the original eye movement feature information, the original eye movement feature $X_{{{\text{tc}}}}$ and the deep eye movement feature $X_{deeper}$ are spliced here to obtain a new eye movement data sequence, denoted as $X_{combine}$, The new eye movement data sequence $X_{combine}$ can retain the original The eye movement feature information also contains the deep eye movement feature information, and the feature dimension included is 87, as shown in Fig. 6. This process can be expressed by the following formula:

4 $$ s\left( {x_{{depper}}^{i} ,x_{{tc}}^{i} } \right) \to x_{{combain}}^{i}  $$

5 $$ S( \cdot ) = [x_{depper}^{i} ,x_{tc}^{i} ] $$

In order to improve the convergence speed and stability of the network, batch regularization can be performed on the new data sequence after splicing^22^,that is the mean $\mu$ and variance $\sigma^{2}$,of all data in the same batch in each dimension are calculated, and in In the process of model training, the scaling coefficient $\gamma$ and the offset coefficient $\beta$, are learned, and the input data *x* is normalized to the output *z*, The formula (6) is as follows:

6 $$ z = \gamma (x - \mu )/(\sigma^{2} + \varepsilon )^{1/2} + \beta $$

where $\varepsilon$ is a very small value taken to prevent the denominator from being zero.

#### LSTM module

**Figure 7:**
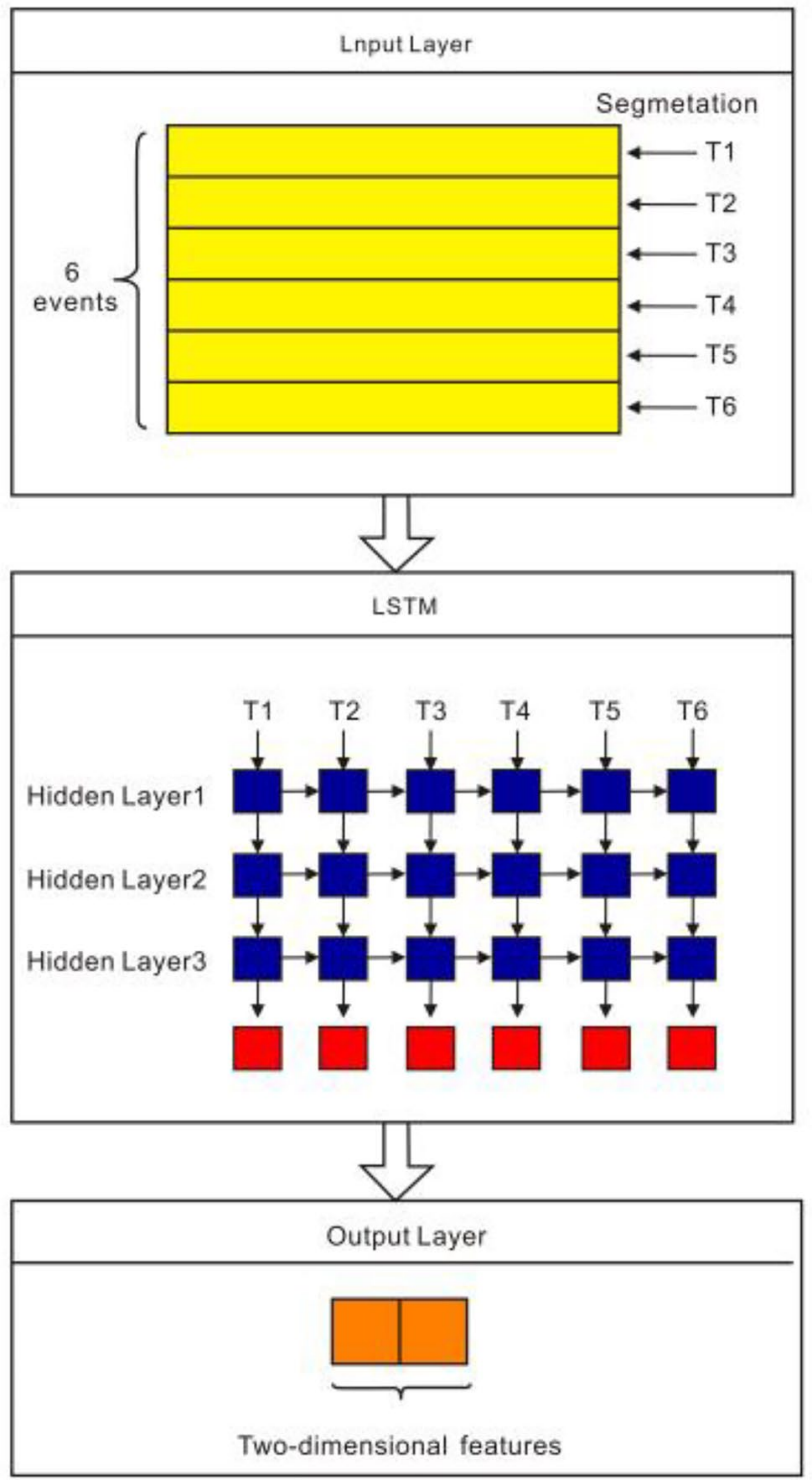
LSTM module.

The spliced data sequence after batch regularization is used as the input of the LSTM neural network, and the input data size is 64 × 6 × 87. In order to increase the sample size and to avoid over-fitting during the training process^23^, the input data is divided into 6 groups according to the dimension of the event sequence. The LSTM network contains 3 hidden layers, and the number of hidden layer units is 128. Therefore, after LSTM processing, a timing feature output with a size of 64 × 6 × 128 will be obtained. This output data is denoted by $X_{tc - feature}$. Finally, we input the timing feature output of the LSTM network into a fully connected layer to obtain a vector of length 2, denoted by $X_{tc - output}$, as shown in Fig. 7.

After inputting $L( \cdot ) = \sigma (\omega_{0} x_{t} +$ into the LSTM module, the time series feature output $L( \cdot ) = \sigma (\omega_{0} x_{t} +$ can be obtained, which can be expressed by the following formula:

7 $$ L(x_{combine}^{i} ) = X_{tc - feature} $$

8 $$ L( \cdot ) = \sigma (\omega_{0} x_{t} + U_{0} h_{t - 1} + b_{0} ), $$

where $\sigma$ is the activation function Sigmoid, $\omega_{0}$ and $U_{0}$ are the weight matrices, and,$b_{0}$ is the bias. Besides, the parameter $h_{t}$ are as follows:

9 $$ h_{t} = L( \cdot ){\text{Tanh}} (c_{t} ), $$

where $c_{t}$ is the current unit state, the unit state at the previous moment is $c_{t - 1}$,and the relationship between them follows

10 $$ c_{t} = f_{t} c_{t - 1} + i_{t} c_{t} , $$

where $c_{t}$ is the instant status of the current unit. The function of $c_{t}$ reads

11 $$ c_{t} = {\text{Tanh}} (\omega_{c} x_{t} + U_{c} h_{t - 1} ), $$

where $\omega_{c}$ and $U_{c}$ are weight matrices. Moreover, $i_{t}$ in Eq. (10) reads

12 $$ i_{t} = \sigma (\omega_{i} x_{t} + U_{i} h_{t - 1} + b_{t} ), $$

where $\sigma$ is the activation function Sigmoid,$\omega_{i}$ and $U_{i}$ are the weight matrices, and $b_{t}$ is the bias. Meanwhile, $f_{t}$ is expressed as

13 $$ f_{t} = \sigma \left( {\omega_{f} x_{t} + U_{f} h_{t - 1} + b_{f} } \right), $$

where $\sigma$ is the activation function Sigmoid, $\omega_{f}$ and $U_{f}$ is the weight matrix, and $b_{f}$ denotes the bias.

### Time-independent eye movement data processing method

For timing-independent eye movement data, it is processed directly through the CNN network, and the processing flow is the same as the above-mentioned CNN module. The output of timing irrelevant eye movement data after passing through the CNN module is denoted by $X_{tic - output}$.

### Spliced data for final classification

Finally, the output vector $X_{tc - output}$ of the timing-related CNN-LSTM module and the output vector $X_{tic - output}$ of the timing-independent CNN module are spliced, and the spliced data is recorded as $x_{final}$, which is then sent to the classifier containing a fully connected layer for the final classification. Using the Softmax function, two probability values are obtained, which represent the probability of the controllers’ current moment of control forgetting.

Using $x_{final}^{i}$ and $y^{i}$ to denote the *i*-th sample and the prediction result respectively, this process can be expressed by the following formula:

14 $$ F(x_{final}^{i} ) \to y^{i} $$

15 $$ F( \cdot ) = Soft\max (FC(x_{final}^{i} )) $$

In this experiment, a total of 100 epochs are trained, and the cross-entropy loss function is used as the loss function of the network. The loss function is

16 $$ Loss(y,class) =  - \log \left( {\frac{{\exp (y[class])}}{{\sum {\exp (y[i])} }}} \right),  $$

where $y$ represents the category probability predicted by the model, $class$ represents the correct category, and $y[i]$ represents the predicted probability of the *i*-th category. In this paper,

### Training algorithm

Adam, a stochastic gradient descent optimization algorithm, is used as the optimizer when training the model^24^. This training algorithm is comprised of the following steps:

1. Initialize the model weights with some starting values. Specifically, the initial learning rate of 0.0001 is adopted, and the Kaiming initialization method is used to initialize the network^25^.
2. For each training iteration:
  - Calculate the gradient of the loss function with respect to the model weights.
  - Calculate the exponential moving average of the gradients and the exponential moving average of the squared gradients.
  - Use these moving averages to adjust the learning rate for the current iteration.
  - Update the model weights using the adjusted learning rate and the gradients calculated in step (b).
3. Repeat steps (2) and (3) until the model has converged or the maximum number of iterations has been reached.

## Result

**Figure 8:**
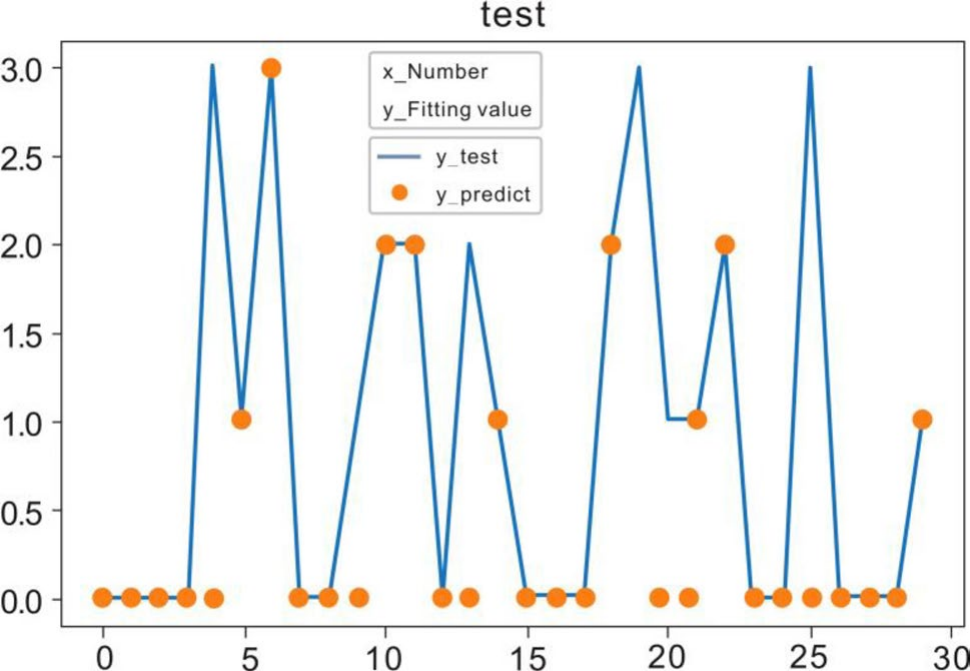
CNN-LSTM prediction effect.

Effective data from 20 subjects are utilized here. To verify the results, we conduct the fivefold cross validation in this study. Specifically, we shuffled and split the dataset into 5 consecutive folds, and then treat each fold as a valid dataset, and the remaining 4 folds as the training sets. The final result is obtained by averaging these five validations. The prediction effect of CNN-LSTM is shown in Fig. 8.

### Comparison of CNN-LSTM neural network and binary logistic regression prediction

In order to evaluate the effectiveness of the CNN-LSTM neural network model in predicting control forgetting, we chose to compare it with the commonly used traditional algorithm binary logistic regression. The prediction accuracy rates of the two methods for control forgetting events are shown in Table 3.

**Table 3:** Comparison of accuracy between CNN-LSTM neural network and binary logistic regression.

Method of prediction	Accuracy
Binary logistic regression	71.3%
CNN-LSTM neural network	79.2%

It can be seen from the comparison results that the commonly used traditional algorithm binary logistic regression has an accuracy of 71.3% in predicting regulatory forgetting events, while the CNN-LSTM model has accuracy up to 79.2%. This is because when the binary logistic regression method is used for prediction, only the basic eye movement features of the controller are used; while the CNN-LSTM neural network works, deeper eye movement features are mined, thereby effectively improving the accuracy of the prediction results.

### Comparison of CNN-LSTM with CNN and LSTM

**Figure 9:**
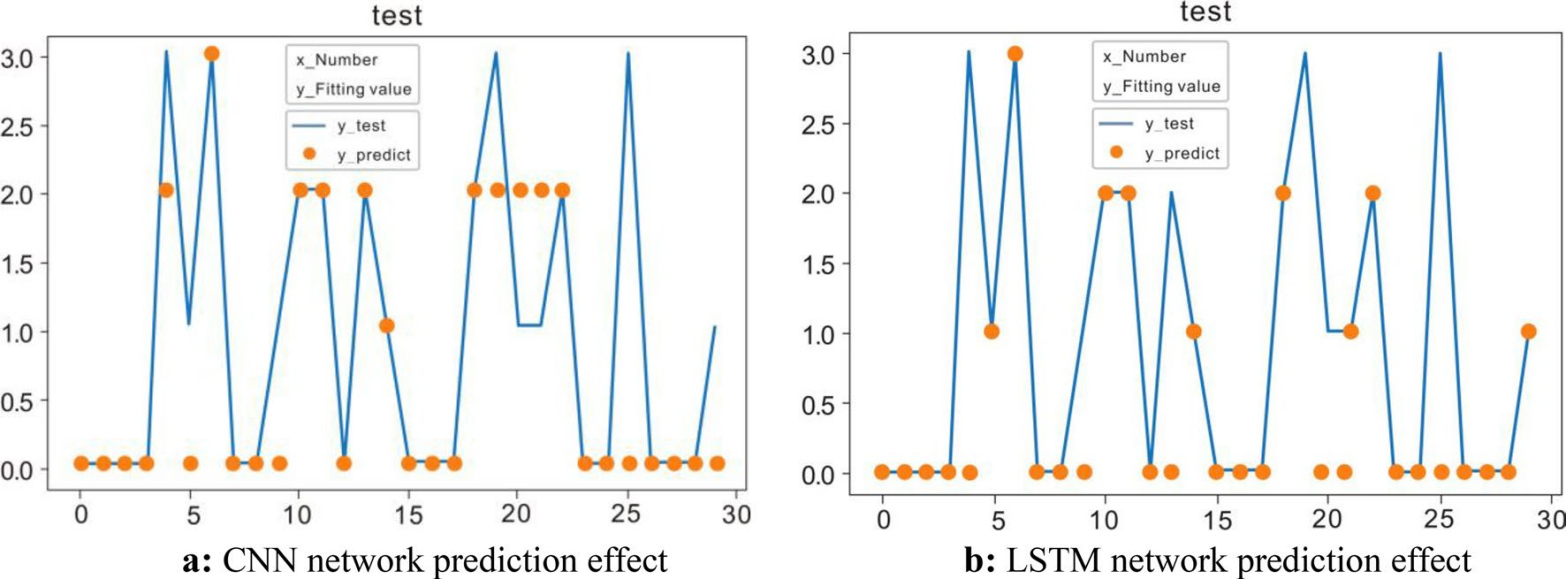
(**a**) CNN network prediction effect (**b**) LSTM network prediction effect.

As is well known, both CNN and LSTM can be used to predict the status of operators. In order to verify the superiority of the CNN-LSTM, it is disassembled for ablation experiments. We remove the CNN part and LSTM part respectively, and conduct a comparative test. Results are shown in Fig. 9, and Table 4 is the comparison result of the accuracy of the ablation experiment.

**Table 4:** Comparison of ablation experiments.

Neural network type	Accuracy
CNN	74.6%
LSTM	75.1%
CNN-LSTM	79.2%

**Figure 10:**
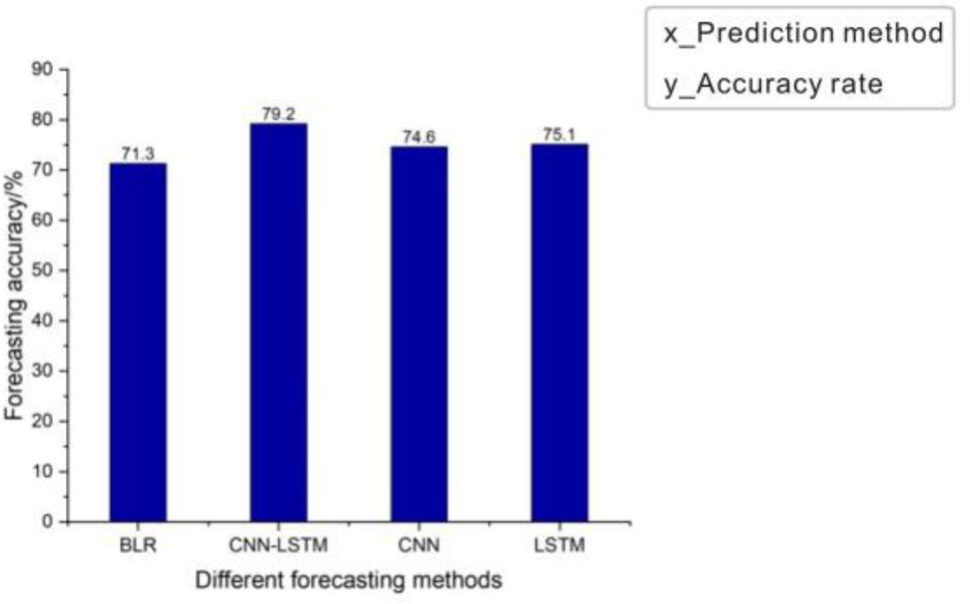
The accuracy rates of different forecasting methods for predicting regulatory forgetting.

The prediction results of these prediction methods are put together for a more intuitive comparison, and the result is shown in Fig. 10.

Results show that compared to the traditional binary logistic regression prediction method, CNN model and LSTM model performs better, but the CNN-LSTM model, combining these two models, outperforms each of them alone in prediction accuracy. This may be because eye movement signal has certain continuity in both time and space. The CNN part can extract the spatio-temporal correlation among eye-movement features to a certain extent, while the LSTM part, which is good at processing time series, has a better effect on extracting time-related eye movement features. Therefore, combination of these two models is effective to deal with eye movement signal.

## Discussion and conclusion

Overall, this paper proposes an innovative method to predict control forgetting. Firstly, we carry out some eye-movement experiments to obtain a feasible data set. Then, the eye-movement data is divided into two types: time-dependent and time-independent features. Finally, the hybrid CNN-LSTM model, as well as the traditional binary regression prediction, CNN and LSTM, is used to predict the occurrence of control forgetting. Results show that CNN-LSTM can not only extract deep features from manual eye movement data, but also retain the original feature-related information. Specifically, compared with the traditional binary regression prediction, the accuracy of this method has been improved by 7.9%; compared with CNN and LSTM, the accuracy of this method has been improved by 4.6% and 4.1% respectively.

It is worth noting that when the difficulty of the control task is almost the same, most of the control forgetting occurs in both the late and early stages of the eye-movement experiment. As well known, during later stages, fatigue of the subjects may leader to regulatory forgetting. Further, here we can also speculate that subjects may spend considerable time in being familiar with the content of experiments or experiment operations, which could result in the early-stage control forgetting. In this sense, to avoid control forgetting, controllers should enter an efficient and excellent working state as soon as possible, and relieve work fatigue in time. Of course, the underlying information hidden in eye-movement data needs further exploring. We hope this work can provide insight into the application of eye movement technology to aviation warning.

## Data Availability

The datasets generated during and/or analyses during the current study are available within the article.
